# Long-Term Trends in Psychiatric Day Hospitalization: A Retrospective Study in Algarve, Portugal

**DOI:** 10.1192/j.eurpsy.2024.1493

**Published:** 2024-08-27

**Authors:** M. T. Viseu, B. Luz, C. Solana

**Affiliations:** Departamento de Psiquiatria e Saude Mental, Centro Hospitalar Universitário do Algarve, Faro, Portugal

## Abstract

**Introduction:**

Current healthcare policies encourage the investment in transition units between hospitalization and outpatient care. Psychiatry day hospitals (DH) serve as partial hospitalization structures that facilitate this transition. The DH at the Faro Unit of the Centro Hospitalar Universitário do Algarve (CHUA), began its activity in 2008, is situated in southern Portugal and provides support to the entire eastern Algarve region (approximately 300,000 people). Its focus is on rehabilitating individuals with severe mental illnesses necessitating multidisciplinary care, with personalized therapeutic plans.

**Objectives:**

We aimed to categorize patients based on diagnoses (primary psychotic disorder, depressive disorder, and others) according to the International Classification of Diseases (ICD-11) and to characterize and compare sociodemographic and clinical data among these three groups.

**Methods:**

A retrospective study spanning from May 2008 to June 2023 was conducted. We assessed sociodemographic, clinical, and epidemiological data of patients undergoing treatment at CHUA Faro Unit’s DH.

**Results:**

Over this period, 541 treatment cycles were carried out to 433 distinct patients, between 18 and 78 years old. Of the total treatments, 38% were for Primary Psychotic Disorder (PPD), 24% for Depressive Disorder (DD) and among the others (39%) the diagnosis of Bipolar Affective Disorder and Personality Disorder predominated . Statistically significant differences were identified among these three groups. The PPD group exhibited a male predominance, whereas DD and others were largely female. Patients in the PPD group were significantly younger (average age of 36 in PPD, 40 in others, and 48 in DD), more likely to be single, and a majority were unemployed (with several patients retired due to disability). No significant differences were noted regarding dropouts, expulsions, or the duration of DH treatment. These results are preliminary, and additional relevant data are being collected and processed.

**Conclusions:**

The diagnostic group’s consideration revealed differences in the social, demographic, and clinical characteristics of patients. These findings offer insights into patient details, enabling the future adaptation of intervention strategies in a more personalized manner.

**Disclosure of Interest:**

None Declared

